# The role of stem cells in pituitary tumour formation

**DOI:** 10.1530/ERC-26-0171

**Published:** 2026-06-18

**Authors:** James Kaufman-Cook, Bence Kövér, Cynthia L Andoniadou

**Affiliations:** ^1^ Centre for Craniofacial and Regenerative Biology, King’s College London, London, United Kingdom; ^2^ Department of Medicine III, Faculty of Medicine Carl Gustav Carus, Technische Universität Dresden, Dresden, Germany

**Keywords:** PitNET, craniopharyngioma, stem cells, SOX2, single-cell RNA-seq

## Abstract

Pituitary tumours are intracranial neoplasms that pose significant clinical challenges due to their potential for recurrence, therapeutic resistance and resultant endocrine dysfunction and mass effects. In the normal anterior pituitary, resident pituitary stem cells (PSCs) contribute to tissue homeostasis and cellular turnover. The extent to which PSCs contribute to tumourigenesis is not known, but an increasing number of studies have been aiming to address this. In this review, we summarise current evidence implicating PSCs and tumour stem-like populations in pituitary tumour biology, including potential roles in tumour initiation, maintenance and progression. We outline practical criteria for defining tumour stem cells and evaluate findings from functional studies of human tumours, emerging single-cell and spatial transcriptomic datasets and murine lineage-tracing models. We also provide a curated overview of published single-cell RNA sequencing studies of pituitary tumours, highlighting reported stem/progenitor populations and transcriptional signatures across tumour subtypes and propose a framework for future genomic analyses. Finally, we discuss the translational implications of these findings, including the potential for targeting stem-like populations and their associated signalling pathways.

## Introduction

The anterior pituitary (AP) is an endocrine organ that orchestrates essential physiological processes, such as growth, reproduction, metabolism and stress response. Dysregulation of AP function through tumourigenesis can have profound systemic consequences, underscoring the importance of understanding both normal physiology and disease mechanisms in the gland.

Pituitary neuroendocrine tumours (PitNETs), historically termed ‘pituitary adenomas’, arise from cells in the AP and are amongst the most common intracranial neoplasms, accounting for 18.1% of all primary brain tumours (1). Their prevalence in the general population varies significantly by detection method, ranging from 1 case per 1,000 for clinically significant tumours to as high as 16.7% (1 in 6) when including incidental findings in autopsy and radiological studies (2, 3). While primarily benign and slow growing, with very rare metastases, these tumours are associated with significant cumulative morbidity. This can result from hormone dysregulation and compression of nearby brain structures, leading to mass effect symptoms, including severe headaches and visual disturbances (4). Local invasion into adjacent anatomical structures is common, with approximately 30–45% of tumours extending into the cavernous or sphenoid sinus (5). A subset of tumours exhibits more aggressive clinical behaviour, with at least 15% classified as clinically aggressive due to resistance to conventional therapies or recurrence during follow-up (5). In recent classifications endorsed by the World Health Organisation, there has been a diagnostic shift from purely histological features of PitNETs to assaying cell lineage-specific transcription factors (including POU1F1, NR5A1 and TBX19), proliferative and clinical features, underscoring the developmental heterogeneity of these tumours (6).

Despite their prevalence, the biological mechanisms that govern PitNET development and account for their diverse clinical behaviour remain only partially understood. In contrast to many other tumour types, recurrent somatic oncogenic mutations are rare in PitNETs (7, 8). While germline variants in genes such as *AIP*, *PRKAR1A*, *MEN1* and *SDHx* underlie a minority of familial cases, most tumours (>95%) arise sporadically and lack clearly defined genetic drivers (7, 8). The relative scarcity of consistent genetic alterations suggests that tumourigenesis may instead reflect dysregulation of cellular identity, lineage specification or tissue homeostasis within the AP, highlighting the need to consider alternative models of tumour formation. A key unresolved question is the cellular origin of PitNETs, with competing hypotheses proposing transformation of differentiated hormone-producing cells, resident stem or progenitor populations or transitional lineage states.

Clinically, PitNETs are classified as functioning or clinically non-functioning based on their hormone secretory activity. Functioning tumours produce excess hormone secretion resulting in well-defined endocrine syndromes, including growth hormone (GH)-, prolactin (PRL)-, adrenocorticotropic hormone (ACTH)- and thyroid-stimulating hormone (TSH)-secreting subtypes. Although gonadotroph tumours may express luteinising hormone (LH) and/or follicle-stimulating hormone (FSH), they are typically clinically non-functioning. Non-functioning tumours (NF-PitNETs) generally do not produce clinically significant hormone excess and usually present through mass effect symptoms and/or hypopituitarism (9).

In addition to PitNETs, craniopharyngiomas (CPs) occur in the same sellar/parasellar region with a prevalence of 0.17–0.2 cases per 100,000 (10). CPs are separated by aetiology into adamantinomatous craniopharyngiomas (ACPs) and papillary craniopharyngiomas (PCP). Despite sharing nomenclature, these tumours are molecularly and histologically distinct. ACP predominantly occurs in children and is characterised by activating mutations in β-catenin (*CTNNB1*), particularly in exon 3, leading to aberrant activation of the WNT signalling pathway (11). In contrast, PCP is more common in adults and is defined by recurrent *BRAF^V600E^* mutations, which drive constitutive activation of the MAPK signalling pathway (11). CPs provide a complementary model to pituitary tumourigenesis, with strong evidence for non-cell-autonomous signalling and niche-driven tumour initiation, particularly in ACP.

Despite advances in surgery (12), radiotherapy (13) and medical management (14), treatment resistance and tumour regrowth remain substantial clinical challenges (5, 6). The ability of pituitary tumours to recur after treatment suggests the presence of intrinsic cellular subpopulations capable of driving disease recurrence. A central unresolved question is whether pituitary tumours are sustained by discrete tumour stem cells (TSCs) or whether they instead arise from aberrant lineage specification and cellular plasticity within differentiated endocrine populations.

The TSC model has emerged as a compelling paradigm to explain tumour initiation, progression, therapeutic resistance and recurrence across multiple malignancies, including both haematological and solid tumours (15, 16, 17). TSCs can be defined as a subpopulation of tumour cells that have the capacity to self-renew, differentiate with multi-lineage potential and enhance tumour-propagating ability *in vivo*, typically demonstrated through serial transplantation experiments (18). In this model, TSCs are proposed to reside at the apex of tumour organisation, generating the heterogeneous bulk of more differentiated tumour cells that constitute a mass. TSCs have been associated with enhanced resistance to cytotoxic therapies and radiotherapy in glioblastoma, endometrial cancer, acute myeloid leukaemia and urothelial carcinoma, suggesting that their persistence may underlie disease relapse (19, 20, 21, 22). This resistance is thought to involve upregulation of DNA repair pathways, increased drug efflux and maintenance of a quiescent state (23, 24). Consequently, therapeutic strategies aimed at selectively targeting and eradicating TSCs have been proposed as a potential route towards more durable and possibly curative treatments for tumours. Supportive evidence now suggests that similar stem-like populations may exist in PitNETs and CPs.

Parallel to the TSC hypothesis, it has been established that the AP contains a population of resident SOX2^+^ pituitary stem cells (PSCs) in both mice and humans. In mouse models, PSCs have the capacity to self-renew and terminally differentiate into the mature hormone-producing and secreting cell types of the AP (25). Importantly, PSCs do not function solely as a reservoir for cell replacement, but act as signalling hubs that orchestrate tissue behaviour through paracrine signalling mechanisms (26, 27). For example, in mouse models, PSCs have been shown to modulate the differentiation and proliferation of adjacent cell populations through the activation of the WNT signalling pathway (27). The identification of PSCs in homeostasis provides a conceptual and experimental foundation for investigating their contribution to pituitary tumourigenesis. Whether this reflects a dysregulation of stem cell identity or reactivation of developmental programmes, through cell-autonomous or non-cell-autonomous mechanisms, can be determined experimentally. However, pituitary tumourigenesis may not conform to classical TSC hierarchy and might instead reflect a spectrum of stem-like states and niche-driven mechanisms.

In this review, we critically evaluate the current evidence supporting the presence of stem and progenitor-like cells in PitNETs and their potential roles in tumour initiation, progression and maintenance. We highlight findings from human studies, genetically engineered mouse models and lineage-tracing experiments, as well as insights gained from single-cell and spatial transcriptomic analyses. To provide a structured overview, we also summarise published single-cell RNA sequencing (scRNA-seq) datasets of pituitary tumours, highlighting stem/progenitor cell proportions and transcriptional signatures across tumour subtypes. Finally, we discuss the therapeutic implications of targeting stem-like populations and outline key unanswered questions that must be addressed to translate these concepts into clinical benefits for patients with pituitary neoplasia.

## Evidence for PSC involvement in human pituitary tumourigenesis

Histopathological analyses have provided indirect evidence for the presence of stem-like cell populations within PitNETs. Immunohistochemical studies of tumour tissues consistently identify expression of stem cell-associated markers, including the transcription factors SOX2, SOX9, OCT4 (encoded by *POU5F1*) and NANOG, the glycoprotein CD133 (encoded by *PROM1*) and the intermediate filament protein NESTIN (encoded by *NES*) (28, 29, 30, 31, 32). These proteins have been reported to exhibit higher expression levels in the tumours than in normal pituitary tissue (23, 29, 30). Notably, one study reported that SOX2^+^ cells exhibited increased proliferative activity in PitNETs compared with normal pituitary, consistent with an activated stem-like state (28). Together, these observations provide strong indirect evidence that TSC-like populations are present and may be functionally relevant in PitNETs.

**Figure 1:**
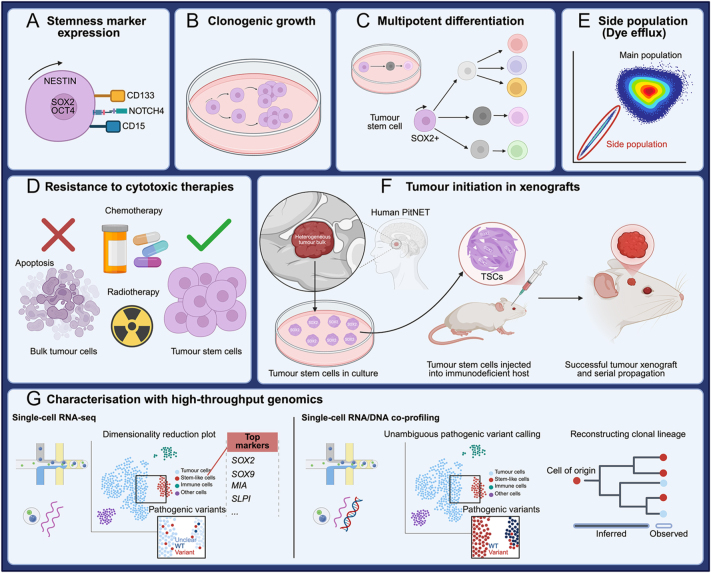
Functional and molecular criteria to identify tumour stem-like cells in human PitNETs. Schematic overview of the principal features used to characterise TSCs in human pituitary neuroendocrine tumours (PitNETs). Candidate TSC populations are identified based on concordant evidence across multiple functional and molecular properties. (A) Expression of stemness-associated markers, including SOX2, CD133, OCT4, NOTCH4, NESTIN and CD15. (B) Clonogenic growth under stem cell-promoting conditions, demonstrating the capacity for sphere formation and self-renewal *in vitro*. (C) Multipotent differentiation potential, whereby undifferentiated tumour cells can generate hormone-producing pituitary cell lineages *in vitro*. (D) Relative resistance to cytotoxic therapies, including chemotherapy and radiotherapy, compared with the bulk tumour cell population. (E) Enrichment of stem-like populations through identification of SP cells characterised by high dye efflux capacity. (F) Tumour-initiating capacity demonstrated by *de novo* tumour formation following transplantation into immunocompromised hosts, ideally with serial propagation to confirm long-term self-renewal. (G) Characterisation with high-throughput genomics. PitNET: pituitary neuroendocrine tumour; TSC: tumour stem cell; WT: wild type, SP: side population.

Building on these findings, direct evidence for TSC-like cells in human PitNETs has emerged from studies isolating and functionally characterising undifferentiated cell populations that satisfy established TSC criteria. These criteria are outlined in Fig. 1 and include expression of stemness-associated markers, such as SOX2, CD133, OCT4, NOTCH4, and NESTIN (Fig. 1A), clonogenic growth under stem cell-promoting conditions (Fig. 1B), multipotent differentiation capacity *in vitro* (Fig. 1C) and resistance to cytotoxic therapies relative to the bulk tumour population (Fig. 1D) (28). Additional enrichment strategies include identification of ‘side population’ (SP) cells based on high drug/dye efflux (Fig. 1E). The most compelling evidence is the demonstration of *de novo* tumour formation following transplantation into immunocompromised hosts, ideally with serial propagation to confirm self-renewal (Fig. 1F) (33, 34). Finally, single-cell genomics approaches have the potential to identify cells with stem-like gene expression profiles and to identify pathogenic variants (Fig. 1G). Recently developed single-cell RNA/DNA co-sequencing platforms could further improve per-cell variant calling and enable reconstructing clonal relationships with high fidelity. Such results could provide strong evidence regarding TSC contribution, yet these experiments have not been performed in the pituitary. As no single criterion definitively establishes TSC identity, concordance across multiple functional and molecular criteria is required.

The first report describing TSC-like population isolation from human PitNETs identified a subpopulation of cells from GH+ and NF-PitNETs capable of forming floating sphere colonies under serum-free, stem cell-promoting conditions (35). These spheres expressed OCT4, NOTCH4, CD133, CD15 and NESTIN and demonstrated self-renewal and multipotent differentiation *in vitro*. Importantly, these cells exhibited greater chemoresistance than their differentiated progeny. Transplantation into immunodeficient mice generated hormone-positive intracranial tumours that could be serially propagated, providing strong evidence of tumour-initiating capacity consistent with a TSC phenotype. Subsequent studies corroborated these findings, showing that CD133^+^/NESTIN^+^ cells isolated from human PitNETs formed floating spheres with multipotent differentiation capacity (36). Upon subcutaneous xenotransplantation, these cells generated synaptophysin-positive tumours, further supporting their tumour-initiating potential. Interestingly, CD133 expression was higher in invasive compared to non-invasive PitNETs, suggesting a potential link to stem cell properties (36). However, another study with a larger cohort of PitNETs did not find an association between CD133 expression and tumour size or recurrence (37). Furthermore, although CD133^+^ cells frequently exhibit stem-like properties, CD133^−^ populations can display comparable clonogenicity, differentiation potential, proliferation, invasiveness and chemoresistance (38, 39). Together, these findings indicate that since CD133 is neither necessary nor sufficient to define pituitary TSC-like cells, it should be interpreted cautiously as a pituitary TSC marker.

Functional enrichment approaches have also utilised SP isolation. SP cells derived from human PitNETs express SOX2 and other stemness markers, form spheres and differentiate into hormone-producing cells *in vitro*, although xenograft formation has often been inefficient (40). In contrast, SP cells expressing SOX2 and CXCR4 from the AtT-20 murine corticotroph cell line demonstrate robust tumourigenicity in immunocompromised hosts (40). In this model, pharmacological inhibition of CXCR4 reduced epithelial–mesenchymal transition (EMT)-associated motility and xenograft growth, implicating this pathway in stem-like tumour behaviour. It is, however, somewhat unexpected for an established cell line of differentiated cells to retain a distinct subpopulation exhibiting a more stem-like phenotype. In parallel, SP cells have also been identified in mouse tumours. SP frequency and SOX2^+^ colony-forming capacity are increased in prolactinomas from *Drd2*^*−/−*^ mice relative to wild-type pituitary controls, further supporting a stem/progenitor contribution to pituitary tumour biology (40).

Further evidence for TSC-like cells was reported in most samples in a large cohort of human PitNETs (41). Rare hormone-negative cells expanded under stem cell-promoting conditions or following CD133^+^ selection and formed fibroblast-free spheroid cultures expressing SOX2, OCT4 and NESTIN (41). These cells exhibited sustained proliferation, self-renewal and multipotent differentiation into hormone-expressing lineages *in vitro*. Notably, they retained functional responsiveness to pharmacological targeting via dopamine (D2R) and somatostatin (SSTR2 and SSTR5) receptors. Although murine xenotransplantation did not yield overt tumours, engraftment into zebrafish embryos induced pro-angiogenic and invasive responses, suggesting functional relevance within a permissive microenvironment (41).

In addition to CD133 and SOX2, CD15 has also been proposed as a TSC-like tumour marker in PitNETs (42). Tumours with high CD15 expression exhibited increased SOX2 levels compared with CD15^Low^ tumours (42). CD15^+^ cells isolated from PitNETs formed spheres in stem cell-promoting conditions and generated tumours in murine xenograft models. Moreover, a higher proportion of CD15^+^ cells was associated with tumour recurrence, suggesting another potential link between stem-like subpopulations and aggressive clinical behaviour.

More recently, TSC-like cells have been isolated from gonadotroph PitNETs (43) and expanded *in vitro* from a series of clinical samples. These were shown to express SOX2, OCT4, NESTIN and CD133 and demonstrated differentiation into FSH-expressing cells in culture. RNA sequencing then compared the TSC-like cells with matched bulk tumour cells identifying over 1,300 differentially expressed genes, which included 843 genes enriched in the TSC-like cells. Amongst the specific hits, several, including *ANXA2*, *PMAIP1* and *SPRY2*, were shown to be upregulated in invasive tumours (43). These data provide both functional and molecular support for a TSC-like subpopulation in gonadotroph PitNETs and highlight candidate mediators of invasiveness, such as *ANXA2* (encoding Annexin A2), as potential therapeutic targets.

Three-dimensional organoid systems provide an additional approach for investigating stem/progenitor populations in PitNETs. Organoids have been generated from dissociated human PitNET samples with high efficiency, including from cryopreserved tissue (28). The resulting cultures expressed several stem cell-associated markers, including SOX2, SOX9, TROP2 (encoded by *TACSTD2*), KRT8/18, CD44 and E-cadherin, while lacking hormone expression, consistent with an undifferentiated phenotype (28). Interestingly, these patient-derived organoids displayed striking histological resemblance to CPs, with defined SOX2^+^ epithelia. Bulk RNA sequencing comparing organoids with matched tumour samples showed enrichment of multiple stem-related genes, further supporting stem-like characteristics in organoids. However, tumour-derived organoids displayed limited expansion capacity and generally failed to propagate beyond the first passage (28). Their limited propagation capacity raises the possibility that current organoid systems preferentially capture epithelial-/stem-like states, rather than faithfully recapitulating tumour hierarchy *in vivo*. This further supports the view that stem-like features in PitNETs may reflect context-dependent cellular states rather than a stable TSC population.

Despite substantial progress, reproducible xenograft formation from human TSC-like populations remains challenging. Although *de novo* tumour initiation and serial transplantation are considered the gold standard for confirming TSC activity, this approach has proven difficult in PitNETs due to their typically benign, indolent nature and the limited proliferative capacity of putative TSC-like populations *in vivo*. This limited proliferative capacity might be explained by senescence, which is a tumour suppression mechanism through permanent cell cycle arrest (44). Indeed, pituitary TSC-like populations in both ACP mouse models and human tumours are characterised by prominent senescence programmes, further supporting a role for senescence-associated mechanisms in ACP pathogenesis (45). Successful engraftment may also depend on microenvironmental cues that are not fully recapitulated in murine hosts. TSC-like marker heterogeneity and reliance on functional enrichment strategies further complicate consistent isolation of TSC-like populations.

Taken together, these studies support the existence of stem-like populations in PitNETs, but also highlight substantial heterogeneity in their phenotype and functional properties. Importantly, while stem-like signatures are consistently detected, compelling functional evidence for robust tumour-propagating capacity remains limited. Rather than representing a uniform entity, TSC-like cells in pituitary tumours are better understood as a spectrum of states influenced by tumour subtype, microenvironment and experimental context. These limitations underscore the need for more precise and functionally validated TSC-like markers, alongside improved *in vivo* and microenvironmentally relevant models.

## Transcriptomic profiling of TSC-like cells in PitNETs

Recent advances in scRNA-seq and spatial transcriptomics have enabled high-resolution interrogation of cellular heterogeneity within pituitary tumours. Unlike earlier described approaches that relied on predefined marker-based isolation, these technologies allow an unbiased identification of transcriptional programmes specific to TSC-like cells. Moreover, these datasets enable the mapping of signalling pathways associated with stem-like states and provide insights into cell–cell communication within the tumour microenvironment.

A major limitation across studies is the inconsistent definition and annotation of TSC-like populations. Similar cell populations are variably annotated as ‘stem-like’, ‘progenitor’, ‘undifferentiated’ or ‘epithelial-like’, often based on overlapping but non-identical marker sets and clustering approaches. For example, *SOX2*^+^ and *SOX9*^+^ cells are explicitly classified as ‘tumour stem-like’ in some datasets but incorporated into broader epithelial or non-endocrine clusters in others. This variability complicates cross-study comparisons and suggests that TSC-like populations may be under-recognised. Despite these differences, a consistent finding is the presence of small populations of cells expressing canonical PSC markers, indicating that TSC-like populations are a common feature of PitNETs. All published single-cell and spatial transcriptomic studies of pituitary tumours to date are summarised in Table 1, where we also highlight reported stem/progenitor populations and associated terminology. Since residual healthy endocrine populations are typically absent from these tumour datasets, the consistent detection of TSC-like cells is unlikely to reflect incidental sampling. If their presence were merely coincidental, a broader representation of normal pituitary cell types would also be expected. Instead, their recurrent identification across studies supports a potential active role for these cells within the tumour compartment.

**Table 1:** Summary of single-cell and spatial transcriptomic studies of pituitary tumours.

Study	Sample description	Total cells/nuclei	Modality	Stem cell cluster reported	Stem cell number/proportion	Stem-associated markers
Cui *et al.* (2021) (46)	23 PitNET samples (multiple subtypes)	2,679	Single-cell RNA-seq	Yes	17	SOX2, SOX9, LHX3
Zhang *et al.* (2021) (54)	4 clinically functional corticotroph tumours	26,126	Single-cell RNA-seq	Yes (3 stem cell clusters)	1,299 (8.3%)	SOX2, SOX9
Asuzu *et al.* (2022) (60)	24 Cushing’s, 1 silent Cushing’s, 3 GH, 3 PRL, 3 NF-PitNETs	27,594	Single-cell RNA-seq	No (folliculostellate cell cluster)	402	SOX2, SOX9, LHX3, HES1, ZFP36L1, ANXA1, HEY1, NFIB, MIA, S100A1
Zhang *et al.* (2022) (51)	5 functioning corticotroph PitNETs, 3 non-functioning corticotroph PitNETs	56,458	Single-cell RNA-seq	Yes	2,054 (3.6%)	SOX2, SOX9, PITX2, LHX3, PAX6, HEY1, MSX1, RFX4, S100B, S100A1/6/10/11, ANXA1/2
Zhang *et al.* (2023) (48)	3 anterior pituitary glands, 21 PitNETs (13 PIT1, 5 TPIT, 2 SF1, 1 null-cell)	6,589	Single-cell RNA-seq	Yes	750	SOX2, SOX9, VIM, S100B, KRT19, TROP2, CLDN4
Lyu *et al.* (2023) (53)	4 PIT1-positive PitNETs	30,374	Single-cell RNA-seq	Yes (3 stem cell clusters)	NR	SOX2, SOX9, LHX3
Lin *et al.* (2024) (62)	3 PIT1 plurihormonal, 1 somatotroph, 7 lactotroph, 2 silent TPIT, 10 SF1 tumours	69,539	Single-cell RNA-seq	Yes (tumour epithelial cells)	NR	SOX2, SOX9, SLPI, MIA, NFIX, S100A1
Matsuda *et al.* (2024) (61)	9 ACPs, 4 PCPs, 1 ciliated craniopharyngioma	192,520	Single-cell RNA-seq	No	N/A	
Potthoff *et al.* (2024) (63)	7 gonadotroph PitNETs, 3 lactotroph PitNETs	37,623	Single-cell RNA-seq	Yes (tumour epithelial cells)	NR	KRT8, KRT18, CLDN4, EGFR, KRT5, KRT7, CLDN5, FGFR1
Wang *et al.* (2024) (56)	2 somatotroph PitNETs and 2 normal pituitary tissues (spatial) + 4 normal pituitary and 16 somatotroph tumours	4,426 spots and 186,892 cells	Single-cell RNA-seq and spatial transcriptomics	Yes	NR	MIA, EPCAM, CLDN4, SLPI
Xu *et al.* (2024) (64)	10 ACP samples	58,081	Single-nucleus RNA-seq	Yes (tumour epithelial cells)	8,825	KRT14, KRT19
Yan *et al.* (2024) (47)	24 PitNET samples + 4 normal pituitary	131,844	Single-cell RNA-seq	Yes	NR	SOX2, SOX9, MIA, SLPI, CLDN4
Ilie *et al.* (2026) (52)	10 somatotroph PitNETs + 4 additional samples with spatial transcriptomics	24,471	Single-cell RNA-seq	Yes	98 (0.4%)	SOX2, SOX9, S100B
Su *et al.* (2025) (49)	13 ACTH, 8 PRL, 3 GH/PRL, 7 GH, 6 TSH, 1 TSH/GH, 6 SGA, 7 SCA, 1 STA, 1 null-cell, 1 FSH adenoma + 3 normal pituitary tissues	35,000 spots and 177,000 cells	Single-cell RNA-seq and spatial transcriptomics	Yes	NR	SOX2, SOX9, SLPI, IGFBP5, AGR2
Wang *et al.* (2025) (56)	10 somatotroph PitNETs + 4 additional samples with spatial transcriptomics	87,862	Single-cell RNA-seq and spatial transcriptomics	Yes	1,396	SLPI, MIA, AGR2, SPARCL1, GPC3, IGFBP5, ID1, ID3, S100A1, SAT1
Zhang *et al.* (2026) (50)	22 PitNET samples (5 PIT1, 7 SF1, 8 TPIT, 2 mixed lineage)	287,051	Single-cell RNA-seq	Yes	NR	SLPI, SCGB1A1, BPIFA1

Overview of published studies using single-cell/nucleus RNA sequencing and spatial transcriptomics, in human pituitary tumours. Reported variables include study design, number of cells/nuclei analysed, sequencing modality, presence of stem cell-like clusters, proportion of putative stem/progenitor cells, associated marker genes and authors’ interpretation of stem-like populations. NR; not reported.

### Evidence for the presence of stem-like populations in PitNET single-cell datasets

An early work by Cui *et al.* identified cells in tumours expressing *SOX2 and SOX9* across multiple subtypes, consistent with a stem-like identity (46). Similarly, Yan *et al.* described an epithelial-/stem-like cluster present in both normal pituitary and tumour samples, characterised by expression of *MIA* alongside *SOX2* and *SOX9* (47). Other analyses have expanded these observations. Zhang *et al.* identified a stem-like population expressing *VIM*, *SOX2*, *KRT19*, *TACSTD2* and *CLDN4* across several PitNET subtypes and confirmed co-localisation of these markers using immunohistochemistry (48). Both immunofluorescence (against SOX2 and Ki67) and scRNA-seq indicated that *SOX2^+^* TSC-like cells are largely non-proliferative, consistent with a quiescent state, typical of a stem-like population. Comparative analyses further demonstrated substantial transcriptional similarities between TSC-like and *SOX2*^+^ cells in the normal AP, suggesting partial recapitulation of physiological PSC states (48). A large multi-subtype study by Su *et al.* analysing 57 PitNETs, similarly, identified clusters of undifferentiated cells enriched for stem-associated genes including *SOX2*, *SOX9*, *SLPI*, *IGFBP5* and *AGR2* (49). Although a substantial proportion of these cells originated from a single tumour, a conserved stemness-associated transcriptional signature, including *WFDC2*, *NFIX*, *IGFBP5* and *TESC*, was observed across tumour lineages, indicating that shared transcriptional programmes may underlie stem-like states in PitNETs. More recently, Zhang *et al.* reported that TSC-like populations exhibit elevated metabolic activity compared to bulk tumour populations. These were enriched for glutathione, ascorbate and aldarate metabolism, as well as oxidative phosphorylation pathways, highlighting cellular metabolism as a potential facet of PitNET stemness that may support differentiation, survival and therapy resistance (50).

Subtype-focused studies have provided additional insight into these TSC-like populations. In corticotroph PitNETs, Zhang *et al.* identified progenitor cells representing approximately 3.6% of tumour cells, expressing developmental regulators such as *SOX2*, *SOX9*, *PITX2*, *LHX3*, *PAX6* and *HEY1* (51). Notably, transcriptional differences were observed between progenitor populations derived from functional and silent tumours. Progenitors from functional tumours were enriched for genes associated with developmental signalling and cell survival, whereas those from silent tumours displayed increased expression of metabolic and proliferative transcripts. Comparable TSC-like populations have also been reported in other tumour subtypes. For example, Ilie *et al.* (2026) identified a small cluster of TSC-like cells in gonadotroph PitNETs, representing approximately 0.4% of captured cells, characterised by expression of *SLPI*, *AGR2*, *MIA*, *SOX2* and *SOX9* (52). Despite differences in tumour identity, TSC-like populations are present across PitNETs.

### Transcriptional heterogeneity and differentiation trajectories

Single-cell analyses also highlight heterogeneity within TSC-like populations. Lyu *et al.* identified three distinct TSC-like clusters within POU1F1-lineage PitNETs, all expressing *SOX2* and *SOX9*, suggesting potential diversity within the TSC-like compartment (53). However, the biological significance of this variability remains unclear and will require validation in larger cohorts with greater cellular depth. Trajectory analyses from this and related studies suggest differentiation pathways from stem-like cells towards more differentiated tumour states, often involving activation of EMT-associated programmes. These transitions are accompanied by progressive loss of stemness markers and epithelial identity genes, alongside acquisition of hybrid epithelial–mesenchymal phenotypes. Complementary work by Zhang *et al.* identified mesenchymal-, epithelial- and endocrine-like progenitor populations in corticotroph tumours and demonstrated that these populations can be maintained in tumouroid culture systems, providing a useful platform for functional interrogation of TSC-like populations (54). The conclusion drawn from trajectory-based analyses should be interpreted cautiously. Stem-like populations are often underrepresented in single-cell datasets and intermediate cellular states may be sparse or entirely missing. This limitation can reduce the robustness of trajectory reconstructions and weaken confidence in the inferred lineage relationships. Ultimately, direct lineage-tracing experiments in mice or single-cell RNA/DNA co-sequencing in humans will be required to validate these proposed differentiation pathways.

### Spatial organisation of stem-like populations

While scRNA-seq provides detailed transcriptional information, it disrupts tissue architecture and may introduce cell type-specific biases. The integration of spatial transcriptomics is, therefore, beginning to provide insights into the spatial organisation of these populations. Studies combining spatial and single-cell approaches have identified TSC-like populations forming discrete clusters throughout tumour tissue, suggesting the presence of localised stem cell niches. For example, Zhang *et al.* identified TSC-like cells in somatotroph PitNETs expressing *SLPI*, *MIA*, *AGR2* and *IGFBP5* that were spatially organised into distinct regions, a finding supported by similar observations from Wang *et al.* (55, 56).

### Clarifying folliculostellate and pituitary stem cell identities

A problem with many single-cell studies remains the inconsistent use of the term ‘folliculostellate (FS) cell’, which originates from morphological descriptions of non-endocrine, star-shaped (‘stellate’) cells forming follicle-like networks in the AP (57, 58, 59). The FS cell does not correspond to a discrete developmentally or molecularly defined cell type. In the normal gland, FS cells have been widely associated with S100 expression and paracrine support functions and overlap with SOX2^+^/SOX9^+^ PSC populations (57, 58, 59). However, this overlap is incomplete and FS cells should not be considered synonymous with PSCs in every tissue context.

In tumour contexts, the continued use of FS terminology becomes particularly problematic. Cells classified as ‘FS-like’ in histological analyses frequently encompass a heterogeneous mixture of non-endocrine populations, including cells expressing PSC markers, stromal cells and infiltrating immune cells. As a result, FS is not a biologically coherent category in tumours but rather a morphology-driven label that collapses distinct cellular types. This ambiguity presents a significant challenge for the interpretation and comparison of single-cell and spatial transcriptomic datasets, where accurate and reproducible cell type annotation is essential.

Recent single-cell studies further illustrate this issue. For example, Asuzu *et al.* identified a PitNET cluster annotated as ‘FS’ that expresses *SOX2*, *SOX9* and *LHX3* (consistent with AP identity), demonstrating substantial overlap with established PSC signatures (60). This molecular similarity suggests that the FS cluster likely includes TSC-like populations rather than representing a distinct FS cell type. More broadly, this supports the view that FS-labelled populations in tumours may obscure bona fide stem/progenitor cells by grouping them under a morphology-based classification.

In addition, immune infiltration is a consistent feature of PitNETs (47, 49, 50) and emerging evidence suggests that interactions between immune, stromal and stem-like populations contribute to the establishment of tumour niches and the regulation of stem cell states. To resolve these challenges, non-endocrine populations in PitNETs should be defined based on molecular identity rather than morphology. Integrating canonical PSC markers with broader transcriptional and epigenetic signatures allows for the discrimination of true stem/progenitor populations from other FS-like, stromal or immune cells. Establishing standardised marker panels and consensus annotation frameworks will be critical for reliably identifying TSC-like populations across studies, ensuring that reported stem/progenitor signatures reflect genuine cell types rather than ambiguous cell morphology.

### Challenges and limitations in defining TSC-like cells

Despite increasing evidence for TSC-like populations in PitNETs, their identification and interpretation remain inconsistent across studies. Dissociation-based approaches may selectively underrepresent fragile or niche-dependent cell populations, potentially biasing detection of TSC-like states. Some scRNA-seq analyses have not explicitly reported stem or progenitor populations (61, 62, 63, 64). For example, Matsuda *et al.* and Lin *et al.* identified tumour epithelial clusters expressing *SOX2* and *SOX9* but did not classify these as TSC-like cells (61, 62), likely reflecting differences in clustering strategies, marker selection or analytical focus rather than true biological absence. Similarly, other studies have described undifferentiated epithelial tumour clusters characterised by keratin and tight junction gene expression (e.g. *KRT* and *CLDN* families) (63, 64), which may represent TSC-like populations that were not formally annotated as stem cells.

Stem-like populations typically comprise a small fraction of tumour cells, limiting statistical power and making cross-study comparisons challenging. Most current evidence also relies on snapshot transcriptional profiling, with limited functional validation of tumour-initiating capacity or lineage dynamics.

Although TSC-like populations are recurrently identified, no PitNET described to date appears to be composed predominantly of undifferentiated stem-like cells. Instead, all tumours exhibit at least partial endocrine differentiation. PitNETs that lack staining for lineage transcription factors (NR5A1, POU1F1, and TBX19), previously thought to represent an undifferentiated state and classified as null-cell adenomas, have since been re-evaluated. Most of these cases show detectable NR5A1 expression and gonadotroph identity, supporting the presence of endocrine differentiation, although true ‘null cell’ tumours may exist (65). Previous reports have also frequently used the legacy terminology of ‘acidophil stem cell pituitary adenomas’ however, these tumours are typically POU1F1+ and PRL+ and there is no convincing evidence that they fulfil contemporary criteria for true stem cell populations (66, 67). Taken together, these observations suggest that there may be diverse TSC-like cells, depending on tumour context and these may have unique properties in terms of influencing the tumour microenvironment.

### Roadmap for future genomic analyses of pituitary tumours

**Figure 2:**
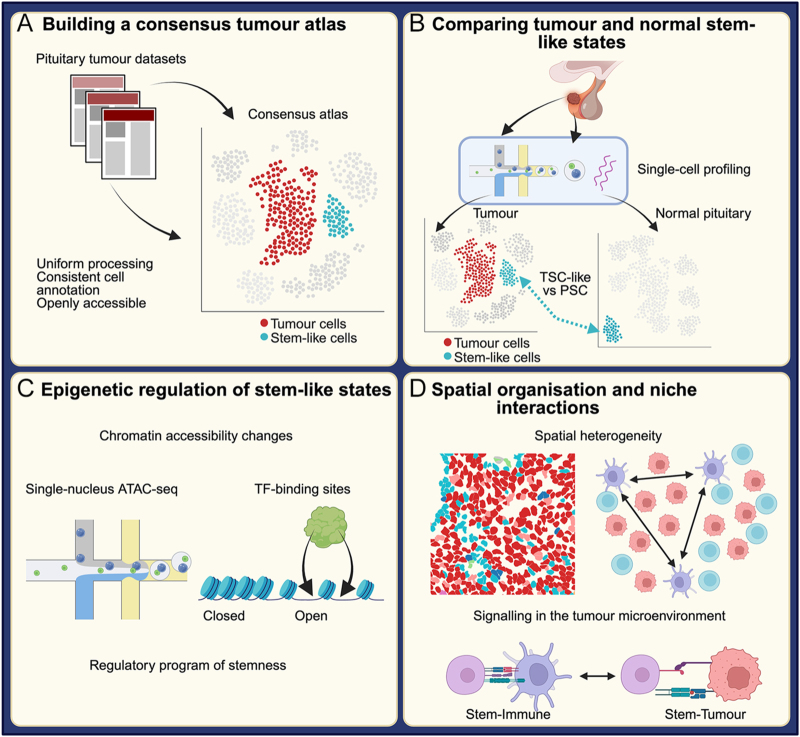
Roadmap for future genomic analyses of pituitary tumours. Schematic outlining proposed avenues for future integrated analyses of genomics data to define the molecular landscape of TSCs and their role in pituitary tumourigenesis. (A) Dataset integration enables consensus tumour atlases with consistent cell state annotation. (B) Tumour vs normal comparisons reveal both shared and tumour-specific stem-like features. (C) Epigenetic profiling identifies regulatory programmes underlying stem-like states. (D) Spatial transcriptomic analyses define the organisation of stem-like cells within tumour niches and highlight interactions with tumour and microenvironmental cell populations. PSC: pituitary stem cell; TSC: tumour stem cell; TF: transcription factor; ATAC: assay for transposase-accessible chromatin.

Future studies will require integration of datasets to enable standardised cell type annotation and robust identification of consensus stem cell signatures across tumour subtypes (Fig. 2A). A similar large-scale approach has recently been demonstrated in the mouse pituitary, where uniform processing of 283 single-cell datasets (>1.3 million cells) enabled cell type-, sex- and age-specific marker discovery (68). Applying comparable strategies to PitNETs would improve statistical power, facilitate cross-subtype comparisons and help define consensus transcriptional programmes of TSC-like cells.

Following integration, analyses should also examine the expression of stem cell markers and associated signalling pathways within the bulk tumour cell populations, as this may uncover the reactivation of stem-like programmes outside classical stem cell clusters. Such phenomena have been observed in phaeochromocytomas and paragangliomas, where chromaffin cells co-express canonical stem markers such as SOX2, with double-positive cells showing active proliferation (69). This raises the possibility that stemness might not be restricted to discrete stem cell compartments but could be reactivated within differentiated tumour cells, potentially contributing to tumour progression. However, technical limitations of single-cell analyses should also be considered in this context. When rare in a tumour, stem-like cells may fail to form discrete clusters and instead be computationally assigned to bulk tumour populations, highlighting the importance of interrogating stem-like gene expression at the single-cell level rather than relying solely on cluster-based classification.

Direct comparison of stem cells from tumour tissue and normal pituitary will be essential to define the molecular changes associated with transformation (Fig. 2B). These approaches will be necessary to determine whether TSC-like populations function as tumour-initiating cells, contribute to tumour maintenance or primarily act as supportive components of the tumour microenvironment.

In parallel, the growing number of bulk RNA sequencing datasets of pituitary tumours can be leveraged using single-cell reference atlases for deconvolution. This enables estimation of stem/progenitor cell proportions across large cohorts and retrospective evaluation of associations between stem-like populations and patient outcomes (70).

Emerging multi-omics approaches are likely to be particularly informative. Single-cell assay for transposase-accessible chromatin (scATAC-seq) will provide insights into the epigenetic regulation and lineage potential within TSC-like populations (Fig. 2C). Enrichment for transcription factor motifs in open chromatin regions might reveal molecular drivers that are missed by RNA abundance alone. In parallel, single-cell RNA-DNA co-profiling approaches, such as DEFND-seq (71), offer the opportunity to directly link transcriptional identity with mutational status at the single-cell level (Fig. 1G). Some of these approaches will also enable resolution of tumour clonality and provide a definitive framework to interrogate the relationship between stem-like cells and tumour evolution. A critical question will be whether TSC-like populations harbour recurrent somatic driver mutations identified in PitNETs (e.g. *USP8* or *GNAS*), thereby establishing whether these cells are part of the neoplastic clone or represent non-neoplastic niche components.

Finally, high-resolution imaging-based spatial transcriptomics approaches (e.g. Xenium, CosMX, and MERSCOPE) should be incorporated into tumour studies to accurately detect rare cell populations, such as TSC-like cells (Fig. 2D). These approaches may better preserve and identify TSC-like populations than dissociation-based single-cell methods, further emphasising the importance of integrating complementary technologies.

## PSC contribution to tumourigenesis in murine models

Most evidence from human PitNET samples derives from clinically detectable tumours, representing relatively advanced stages of disease. As a result, these studies cannot readily determine whether TSC-like populations act as tumour-initiating cells or arise later during tumour progression. Therefore, genetically engineered mouse models, particularly when combined with lineage-tracing approaches, provide a complementary system to interrogate the cellular origin and development of pituitary tumours. Studies using these models have revealed that PSC-driven tumour formation may occur through direct transformation of stem cells themselves (72, 73) or non-cell-autonomous paracrine effects in which mutated stem cells promote tumourigenesis in surrounding cells by establishing a pro-tumourigenic microenvironment (25, 74, 75, 76, 77). Importantly, these mechanisms are not mutually exclusive and may operate concurrently within the same tumour.

### Cell-autonomous PSC transformation

Following initial work that identified the activity of the YAP/TAZ signalling pathway in healthy PSCs and PitNET samples, several mouse models have been generated to characterise this pathway (78, 79, 80). Deletion of *Lats1*, encoding a kinase that negatively regulates the activity of the YAP/TAZ pathway, leads to constitutive YAP/TAZ activation (72). Deleting *Lats1* from the onset of pituitary organogenesis via *Hesx1-Cre* resulted in the development of aggressive non-functioning pituitary tumours predominantly composed of SOX2^+^ cells and exhibiting features of squamous cell carcinoma (72). Conditional deletion of *Lats1* specifically in PSCs using the inducible *Sox2-CreERT2* driver was sufficient to induce tumour formation, confirming that PSCs can serve as the direct cell-of-origin in this context, conforming to the traditional TSC paradigm. Moreover, elevated YAP/TAZ signalling has been associated with poorly differentiated pituitary tumours and repression of differentiation (80). These findings implicate YAP/TAZ dysregulation in PSCs as a potential driver of pituitary tumourigenesis with therapeutic potential.

The cyclin-dependent kinase inhibitor p27 provides a second example of PSC-mediated tumourigenesis. Mice lacking p27 developed pituitary hyperplasia and intermediate lobe tumours, accompanied by an expansion of SOX2^+^ cells within the epithelium (81). Haploinsufficiency of *Sox2* markedly attenuated tumour development, implicating SOX2 as a critical mediator of transformation. Conditional genetic experiments demonstrated that SOX2 is required within *p27*^*−/−*^ melanotrophs for their transformation into tumour cells (73). Deletion of *Sox2* regulatory region 2 (*Srr2*), a target of P27-mediated repression, further confirmed that de-repression of SOX2 drives tumourigenesis (73). Single-cell transcriptomic analyses revealed activation of a SOX2-dependent MAPK programme within stem cells, suggesting that PSCs promote tumourigenesis through expansion and by establishing a pro-tumourigenic signalling environment (73).

### Non-cell-autonomous PSC-driven tumourigenesis

The first direct demonstration of PSC-driven, non-cell-autonomous tumourigenesis was provided by a mouse model of ACP. Inducible activation of oncogenic β-catenin specifically in SOX2^+^ PSCs led to the formation of tumours that recapitulated key histological features of human ACP, including characteristic ‘whorl-like’ clusters and nucleocytoplasmic β-catenin accumulation (25). Lineage tracing revealed that while the β-catenin accumulating clusters originated from the SOX2-targeted PSCs, the majority of the tumour mass consisted of non-targeted cells. This finding demonstrated that genetically altered PSCs do not directly contribute to the tumour bulk in a cell-autonomous manner. Instead, mutant PSCs function as paracrine signalling hubs, secreting WNTs, SHH, FGFs and BMPs to activate core developmental and stemness-associated pathways in neighbouring cells, thereby promoting proliferation and transformation. Molecular analysis of PSCs with activation of oncogenic β-catenin revealed activation of a senescence-associated secretory phenotype (SASP), including interleukins (ILs), matrix metalloproteinases (MMPs) and chemokines (e.g. CXCLs) (45). Human ACP samples displayed analogous senescent β-catenin-accumulating clusters, supporting the translational relevance of the model. Notably, genetic attenuation of senescence and SASP responses in mice significantly reduced tumour-initiating capacity, highlighting senescent PSCs and their secretome as potential therapeutic targets to prevent tumour initiation and progression in human ACP. 

Early progenitor dysregulation models provide complementary developmental evidence for non-cell-autonomous PSC involvement. Overexpression of *PROP1*, a critical transcription factor for early AP development, in embryonic pituitary progenitors resulted in the formation of pituitary tumours (75). However, *PROP1* expression was not maintained in the tumour cells themselves. This suggests that early progenitor/stem cell dysregulation may initiate tumourigenesis via paracrine or niche-mediated mechanisms rather than serving as a sustained oncogenic driver. Similarly, transgenic overexpression of pituitary tumour-transforming gene 1 (*PTTG1*) in early endocrine progenitors using *αGSU-Cre* led to focal plurihormonal hyperplasia and hormone-secreting microadenomas (76). These observations highlight that expansion of multipotent progenitor populations and disruption of lineage fidelity contribute to early tumourigenic events.

Conditional activation of MAPK pathway components (*Braf^V600E^* or *Kras^G12D^*) during pituitary development further supports a role for stem/progenitor dysregulation in tumourigenesis. In these models, MAPK activation resulted in hyperplasia, abnormal gland morphogenesis, impaired lineage commitment and a marked expansion of SOX2^+^ cells, accompanied by increased clonogenic potential (77). These findings indicate that MAPK overactivation shifts the balance within the stem/progenitor compartment away from differentiation and towards proliferation. Consistent with this concept, PCP, which frequently harbours *BRAF^V600E^* mutations, contains a well-defined epithelium of SOX2^+^ cells lining fibrovascular cores. These SOX2^+^ cells retain sustained proliferative capacity, representing the major proliferating cell population in PCP (77). These observations raise the possibility that MAPK-driven activation or expansion of PSC populations may contribute to PCP pathogenesis. However, definitive demonstration of a PSC cell-of-origin will require postnatal, SOX2-restricted targeting of *Braf^V600E^* to determine whether mutant PSCs can initiate tumour formation.

### Tumour-propagating stem-like populations

In addition to models in which PSCs function as the cell-of-origin, evidence also suggests that stem/progenitor populations may support tumour maintenance and propagation in established mouse pituitary tumours. In spontaneous pituitary tumours arising in *Rb*^+/−^ mice, Nestin-GFP^+^ cells expressing stem-associated markers including *Sox2*, EpCAM and cytokeratin 8 were observed surrounding tumour nodules. These lacked hormone expression, consistent with an undifferentiated compartment associated with tumour initiation and growth, although functional tumour-propagating capacity was not directly assessed (82). In the same mouse model, *Sca1*^+^ cells from pituitaries formed spheres under serum-free conditions and expressed stem-associated markers including *Sox2*, Nestin and CD133, while lacking hormone expression (83). *Sca1*^+^ cells exhibited increased clonogenicity, reduced differentiation marker expression and greater proliferative potential (83). Upon transplantation into immunodeficient mice, these cells generated larger, plurihormonal tumours at higher frequencies than *Sca1*^−^ populations (83). These data provide direct evidence for tumour-propagating stem/progenitor cells within the mouse pituitary.

Together, studies of PSC transformation and tumour-propagating stem-like cells in mice provide compelling experimental support for PSC involvement in pituitary tumourigenesis. However, these insights derive from a relatively small number of models in which stem cell contributions were specifically examined. Many historic genetically engineered mouse models of PitNETs were developed without formally interrogating the cellular origin of tumours, leaving potential stem/progenitor involvement unresolved (84). Revisiting these systems using conditional stem/progenitor-specific *Cre* drivers (e.g. *Sox2^CreERT2^* (85)) and lineage-tracing strategies (e.g. YFP reporters or *Rosa26^mTmG^*) could clarify the role of PSCs in tumour initiation and progression, providing important mechanistic insight and potentially revealing cellular vulnerabilities with therapeutic relevance. In addition, genetically barcoded mouse models (e.g. PolyloxExpress and LARRY) of pituitary tumours could enable both measuring RNA and reconstructing clonality from single cells (86, 87). Such methods will be favoured over RNA/DNA co-profiling in tumours with low mutational burden. In addition to lineage tracing, measuring the transcriptional history of cells, with approaches such as ‘TimeVault’, can be informative to understand tumour dynamics and drug responses (88). Overall, these models indicate that PSCs can contribute to tumourigenesis through multiple, context-dependent mechanisms, challenging a single linear model of tumour initiation.

## Clinical implications

The emerging evidence for TSC-like populations has several important implications for the clinical management of pituitary tumours. The presence of SOX2^+^ stem-like cells, which may range from relatively quiescent to highly clonogenic depending on context and which retain therapy resistance and multi-lineage differentiation capacity, provides a potential biological explanation for tumour persistence and recurrence in a subset of PitNETs despite apparently effective surgical and medical therapy. Single-cell studies have identified transcriptional programmes such as NOTCH signalling activation, EMT pathways and metabolic adaptations. In parallel, experimental models have identified signalling pathways such as YAP/TAZ in non-secreting pituitary tumours including CPs, and MAPK signalling in PCPs, as potential therapeutic vulnerabilities that may contribute to tumour propagation and treatment resistance. In gonadotroph-PitNETs, *ANXA2* has been identified as a mediator of tumour aggressiveness, highlighting an additional potential therapeutic target (43). This is of interest as ANXA2 has emerged more broadly as a potential therapeutic target in cancer and autoimmune disease contexts, with accumulating evidence suggesting roles in reducing tumour growth, overcoming therapy resistance and modulating inflammatory processes (89).

Murine lineage-tracing studies further suggest that stem cells may influence tumourigenesis through both cell-autonomous transformation and non-cell-autonomous mechanisms, including paracrine signalling from senescent or oncogene-activated PSCs that establish pro-tumourigenic niches. These findings raise the possibility that effective therapy may require targeting not only differentiated tumour cells but also the supportive signalling networks and microenvironmental interactions that sustain TSC-like populations. Clinically, markers associated with these populations such as SOX2, CD15, CXCR4 or stemness-related transcriptional signatures may ultimately aid in identifying tumours with increased invasive potential or risk of recurrence, although their value as predictors of invasion and aggressiveness requires validation in large and diverse patient cohorts. In parallel, emerging experimental platforms including patient-derived tumouroids and organoid systems provide opportunities to functionally interrogate stem-like tumour compartments and test targeted therapies.

## Conclusions and future directions

**Figure 3:**
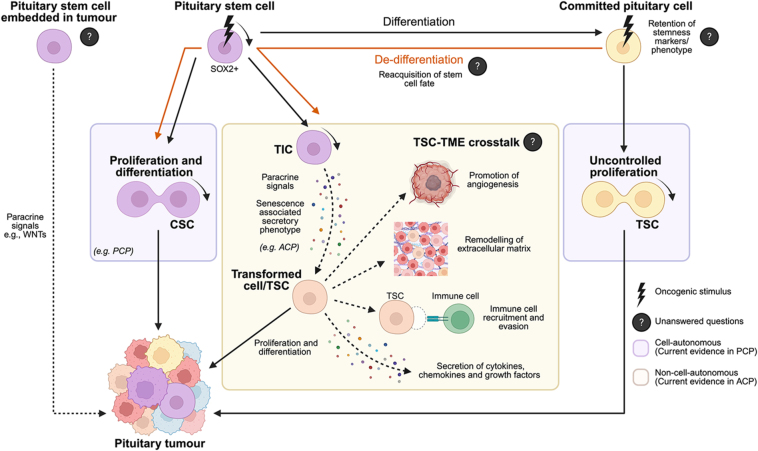
Stem cell models of pituitary tumourigenesis. Schematic illustrating the potential roles of PSCs and TSC-like cells in tumour formation. Under oncogenic stimuli, stem cells can drive tumourigenesis through cell-autonomous mechanisms by proliferating rapidly, differentiating and directly giving rise to tumours, in a classic cancer stem cell (CSC) paradigm (purple box). Thus far, this has been demonstrated in SOX2+ cells harbouring mutations in *BRAF^V600E^* in human PCP. Tumourigenesis can also occur via non-cell-autonomous mechanisms (yellow box), where paracrine signals or a senescence-associated secretory phenotype from a transformed tumour-initiating cell (TIC) promotes proliferation and/or transformation of other cells into TSCs or TSC-like cells. Thus far, this has been demonstrated only in ACP. Whether TSC-like cells contribute to tumourigenesis through either cell-autonomous or non-cell-autonomous mechanisms in pituitary neuroendocrine tumours (PitNETs) remains to be determined. PSCs exposed to oncogenic stimuli that become lineage-committed may retain stem-like characteristics, marker expression or proliferative potential, contributing to tumour formation as TSCs. Conversely, and still unconfirmed, committed endocrine cells may undergo de-differentiation in response to oncogenic signals, acquiring stem-like properties and promoting tumourigenesis through either cell-autonomous or non-cell-autonomous pathways, as TSCs. These populations may also influence tumour progression by modulating the tumour microenvironment. Finally, healthy PSCs may become trapped within the expanding tumour mass and contribute to tumour maintenance or progression through paracrine signalling mechanisms known to regulate the proliferation of neighbouring cells, including the secretion of factors such as WNT ligands. PSC: pituitary stem cell; CSC: cancer stem cell; TIC: tumour-initiating cell; TSC: tumour stem cell; TME: tumour microenvironment. ACP: adamantinomatous craniopharyngioma. PCP: papillary craniopharyngioma.

Collectively, evidence from human tumours, single-cell and experimental models supports a revised framework for pituitary tumourigenesis, in which stem and progenitor-like populations play context-dependent and multifaceted roles. Rather than a single linear hierarchy, tumour development may arise through multiple, potentially co-existing trajectories (Fig. 3). These findings support a hybrid model of pituitary tumour organisation integrating stem cell clonal contribution, cellular plasticity and niche-driven signalling. These trajectories include direct transformation of PSCs, non-cell-autonomous tumour initiation mediated by paracrine signalling from oncogene-activated or senescent PSCs, and potentially the reactivation of stem-like states within differentiated endocrine cells. The contribution of these distinct facets of TSC activity likely varies amongst tumour types (ACPs, PCPs, and PitNETs) and this variation may underlie differences in their aetiologies. In ACP, TSCs likely contribute predominantly through non-cell-autonomous mechanisms, consistent with mouse models in which SOX2^+^ PSCs with oncogenic mutations promote tumour formation indirectly. In contrast, PCPs may arise directly through cell-autonomous mechanisms, supported by the identification of Ki-67^+^ mutation-harbouring SOX2^+^ cells within tumours. The role of stem cells in PitNETs remains less clearly defined. While many mouse models demonstrate direct tumour-initiating potential, bulk and single-cell transcriptomic studies in human tumours have not identified tumours composed solely of SOX2^+^ cells. This suggests that, in humans, stem cells may instead contribute through several alternative mechanisms, including paracrine promotion of tumourigenesis. Alternatively, stem cells may be recruited into the tumour microenvironment, where they may either promote transformation through paracrine signalling, proliferate and contribute to the tumour mass or represent passively entrapped bystander populations. In either case, stem cells may secrete ligands that facilitate or fail to facilitate transformation. The precise non-cell-autonomous mechanisms by which TSCs might contribute to PitNET pathogenesis remain to be fully elucidated and may offer new therapeutic opportunities.

Single-cell and spatial studies reinforce the TSC model by consistently identifying rare, largely quiescent SOX2^+^ populations enriched for developmental, EMT-associated and metabolic programmes. These cells likely represent a reservoir of tumour-propagating potential, while their spatial organisation into discrete niches highlights the importance of local signalling environments. However, a central challenge remains regarding the precise definition of TSC-like cells, given the historical use of morphology-based FS terminology and inconsistent annotation of non-endocrine populations across studies. Importantly, stemness in PitNETs may not be restricted to a fixed cell population but instead may represent a transient and reversible cellular state, further complicating their identification and functional characterisation.

While mouse models provide strong mechanistic support for the TSC framework, a definitive validation in human PitNETs remains limited by the intrinsic biological features of these tumours, including low proliferation and senescence-associated programmes that restrict successful xenograft modelling. A key unresolved question is how these stem-like populations relate to clinically aggressive behaviour. While most PitNETs are indolent, a subset exhibits invasion, recurrence and treatment resistance and rare cases progress to carcinoma. Whether these phenotypes are driven by specific TSC-like populations or microenvironmental interactions remains unclear but represents a critical area for future investigation with direct clinical relevance.

Looking forward, integration of multi-modal datasets at scale will be essential to resolve these challenges. Advances in single-cell multi-omics, spatial transcriptomics and lineage tracing will enable more precise mapping of stem-like populations, their lineage relationships and their roles in tumour evolution. Computational approaches, including machine learning, will support harmonisation of heterogeneous studies and defining more consistent stemness-associated signatures across tumour subtypes. Such approaches may also aid in linking cellular identity with mutational status and spatial organisation, providing insight into tumour hierarchy and microenvironmental interactions. In parallel, analysis of clinical datasets, including imaging, histopathology and molecular profiles, may facilitate the identification of stemness biomarkers associated with tumour behaviour, recurrence risk and therapeutic response.

From a translational perspective, the TSC framework highlights multiple potential therapeutic entry points. Targeting stem cell maintenance pathways (e.g. YAP/TAZ, WNT, and MAPK), disrupting tumour–microenvironment interactions or modulating senescence-associated signalling networks (e.g. through senolytics and/or senomorphics) may complement existing therapies that primarily target differentiated tumour cells. Senescence emerges as a particularly interesting point of intervention, as the age-dependent accumulation of senescent cells and inflammatory signalling may contribute to the increasing prevalence of pituitary tumours with age, despite the relative lack of recurring driver mutations. In this context, inflammaging and cellular senescence are likely interconnected rather than independent processes within the ageing pituitary microenvironment. Indeed, aged mouse PSCs display a SASP-like inflammatory transcriptional profile enriched for IL-6-related signalling pathways (68, 90). Senescent cell clusters in ACP also secrete inflammatory mediators including IL-6 and CXC chemokines (45), suggesting convergence between senescence-associated secretory programmes and age-related inflammatory signalling. However, therapeutic targeting of senescent cells should be approached cautiously, given the established protective role of senescence in preventing oncogenic transformation. Moreover, evidence supporting a tumour-promoting role for senescent TSC-like populations is currently strongest in ACP, whereas its relevance to PitNETs and PCPs remains speculative.

**Figure 4:**
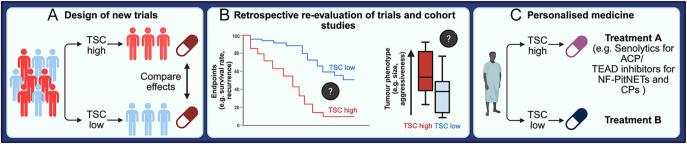
Clinical implementation of TSC-based patient stratification and personalised therapy. (A) Classifying patients into TSC-high and TSC-low groups would enable identification of stem cell-specific effects of new treatments. (B) Retrospective analysis of existing trials and large cohorts by quantifying stem cell abundance in banked samples could reveal associations with various endpoints and clinical phenotypes. (C) TSC abundance measured in individual patients could inform personalised treatment options in the future. These insights might be disease-dependent and apply differently to PCPs, ACPs and PitNETs. TSC: tumour stem cell.

To accelerate the development of further therapies, data-driven patient stratification approaches (e.g. through newly discovered marker genes) could improve the design of clinical trials (Fig. 4A). In addition, reanalysis of large cohort studies and trials, where tissue bank samples are available for staining, could reveal retrospective associations with patient outcomes and stem cell abundance (Fig. 4B). These efforts will pave the way towards targeting stem-like populations as a novel precision medicine strategy in pituitary tumours (Fig. 4C), although the optimal therapeutic strategies will likely differ between PitNETs, ACPs and PCPs, reflecting their distinct underlying aetiologies.

In summary, the TSC paradigm provides a unifying but evolving framework for understanding pituitary tumour biology. Current evidence supports a model in which tumour initiation and progression arise from the interplay between stem cell hierarchies, cellular plasticity and microenvironmental signalling. However, definitive identification of tumour-initiating populations in human PitNETs and clarification of their clinical significance remain key priorities. Addressing these challenges through integrated experimental, computational and clinical approaches will be critical to determine whether targeting stem cell dynamics can be translated into improved outcomes for patients with pituitary neoplasia.

## Declaration of interest

The authors declare that there is no conflict of interest that could be perceived as prejudicing the impartiality of the work reported.

## Funding

The authors were funded by the Medical Research Council (MRC) (grants APP40962 and MR/T012153/1 to CLA) and the Deutsche Forschungsgemeinschaft (DFG, German Research Foundation) (Project no. 314061271, TRR 205: ‘The Adrenal: Central Relay in Health and Disease’ and Project no. 288034826, IRTG 2251: ‘Immunological and Cellular Strategies in Metabolic Disease’ to CLA). JKC was funded by King’s College London and MRC as part of the Doctoral Training Partnership in Biomedical Science. BK was funded by the Wellcome Trust as part of the ‘Advanced Therapies for Regenerative Medicine’ PhD Training Programme (218461/Z/19/Z).

## Author contribution statement

JKC drafted the manuscript and generated the figures. BK contributed to drafting specific sections of the manuscript and assisted in figure generation. CLA supervised the study, guided the writing and secured funding. All authors reviewed, edited and approved the final manuscript.
